# Haem oxygenase protects against thrombocytopaenia and malaria-associated lung injury

**DOI:** 10.1186/s12936-020-03305-6

**Published:** 2020-07-01

**Authors:** Isaclaudia G. de Azevedo-Quintanilha, Isabel M. Medeiros-de-Moraes, André C. Ferreira, Patrícia A. Reis, Adriana Vieira-de-Abreu, Robert A. Campbell, Andrew S. Weyrich, Patricia T. Bozza, Guy A. Zimmerman, Hugo C. Castro-Faria-Neto

**Affiliations:** 1 Laboratório de Imunofarmacologia, Instituto Oswaldo Cruz, Fundação Oswaldo Cruz, Rio de Janeiro, RJ Brazil; 2 Universidade Iguaçu, Nova Iguaçu, RJ Brazil; 3 Division of Endocrinology, Metabolism, and Diabetes, University of Utah School of Medicine, Salt Lake City, UT USA; 4 Department of Internal Medicine and Program in Molecular Medicine, University of Utah, Salt Lake City, UT USA

**Keywords:** Acute lung injury, Acute respiratory distress syndrome, Inflammation, Malaria, Platelets, Heme oxygenase 1

## Abstract

**Background:**

Malaria-triggered lung injury can occur in both severe and non-severe cases. Platelets may interact with parasitized erythrocytes, leukocytes and endothelium. These interactions can lead to microvessel obstructions and induce release of inflammatory mediators. Induction of the haem oxygenase enzyme is important in the host’s response to free haem and to several other molecules generated by infectious or non-infectious diseases. In addition, an important role for the haem oxygenase-1 isotype has been demonstrated in experimental cerebral malaria and in clinical cases. Therefore, the present work aims to determine the influence of haem oxygenase in thrombocytopaenia and acute pulmonary injury during infection with *Plasmodium berghei* strain NK65.

**Methods:**

C57BL/6 mice were infected with *P. berghei* and analysed 7-10 days post-infection. For each experiment, Cobalt Protoporphyrin IX/CoPPIX or saline were administered. Bronchoalveolar lavage fluid was used for total and differential leukocyte count and for protein measurement. Lungs were used for histological analyses or for analysis of cytokines and western blotting. The lung permeability was analysed by Evans blue dye concentration. Platelet-leukocyte aggregate formation was assayed using the flow cytometer.

**Results:**

*Plasmodium berghei* NK65 infection generated an intense lung injury, with increased levels of inflammatory mediators, oedema, and cell migration into the lung. *Plasmodium berghei* infection was also accompanied by marked thrombocytopaenia and formation of platelet-leukocyte aggregates in peripheral blood. Treatment with the HO-1 inducer cobalt protoporphyrin IX (CoPPIX) modified the inflammatory response but did not affect the evolution of parasitaemia. Animals treated with CoPPIX showed an improvement in lung injury, with decreased inflammatory infiltrate in the lung parenchyma, oedema and reduced thrombocytopaenia.

**Conclusion:**

Data here presented suggest that treatment with CoPPIX inducer leads to less severe pulmonary lung injury and thrombocytopaenia during malaria infection, thus increasing animal survival.

## Background

According to the World Health Organization (WHO) malaria remains a major public health problem. In 2018, there were an estimated 220 million cases of malaria in 87 countries, with estimated number of malaria deaths stood at 405,000 [1]. The malaria symptomatic triad is formed by the association of fever, chills and headache. The non-specificity of these symptoms leads to a delay in seeking medical attention and to delayed diagnosis and treatment. This scenario is a recipt to serious clinical complications of the disease, such as metabolic acidosis (mainly respiratory distress), malaria associated acute respiratory distress syndrome (MA-ARDS), cerebral malaria (MC) and severe malaria anaemia (SMA) [2]. MA-ARDS, common in infections caused by *Plasmodium falciparum* and *Plasmodium vivax* [3]. The most common manifestation of pulmonary malaria is pulmonary oedema, the accumulation of alveolar fluid. In addition, MA-ARDS involves accumulation of monocytes and macrophages in the alveoli and an intravascular inflammatory response in pulmonary microvessels. Pulmonary oedema is triggered by increased permeability of the alveolar-capillary membrane, leading to loss of intravascular fluid, with accumulation of macrophages followed by intravascular inflammatory response. The immune response generated in the lung can also lead to airway obstruction [4–7].

In malaria there is also a marked thrombocytopaenia, which is rarely accompanied by haemorrhage, so it is often not considered as one of the signs of severity; however, a case study of children with falciparum malaria found that thrombocytopaenia has a positive correlation with death [8]. Platelets are discoid-shaped anucleated cells that have a diameter of 1 to 3 μm. A healthy individual possesses 150–350 × 10^9^ platelets/L. In addition to their role in thrombosis and haemostasis, platelets participate in other pathophysiological processes including inflammation, atherogenesis, host defense, tumor growth and metastasis. Activated platelets have various mechanisms through which they can deliver signals to leukocytes, endothelial cells and other target cells. The best known is the rapid secretion of soluble mediators (for example von Willebrand factor, CXCL4 (PF4), CCL5 (RANTES)) with endocrine-paracrine signaling activity [9–11].

When platelets are activated in pathological situations they may contribute to the breakdown of the endothelial barrier, leading to fluid leakage and oedema formation. Aggregate of activated platelets and each of the major classes of leukocytes have been reported in clinical samples and/or inflammatory models [12–14]. It is believed that the pathogenesis of malaria-induced thrombocytopaenia is multifactorial and involves bone marrow suppression, antibody-mediated platelet destruction, platelet phagocytosis, adhesion to activated endothelium and interactions with parasitized erythrocytes and leukocytes leading to sequestration of these aggregates and oxidative stress generated during parasite growth [15–17].

Pathogen-associated molecular patterns (PAMPs), including glycosylphosphatidylinositol (GPI) [18] and haemozoin [19] may mediate pathologic events triggered by plasmodial parasites. In addition, haem may be a key molecular agonist in malaria. During malaria infection haemoglobin is used as a source of nutrient for replication by the *Plasmodium* sp. Digestion of haemoglobin leads to haem release. Several studies point to haem as capable of generating inflammation similar to malarial infection [20]. Free haem accumulates on the plasma of children with cerebral malaria triggered by *P. falciparum*, suggesting that it may have a pathological effect on human malaria [21]. Free haem is metabolized by haem oxygenase-1 (HO-1) leading to the release of three by-products, equimolarly, the free iron ion (Fe^2+^), carbon monoxide (CO) and biliverdine [22]. The role of HO-1 enzyme has already been studied in experimental cerebral malaria models [23] and in the pathogenesis of non-encephalic forms of severe malaria [24]. Pena et al. and Pereira et al. described a protective effect for HO-1 in malaria-induced lung injury [25, 26].

Pulmonary malaria in patients develops before, during or after initiation of treatment [27], when release of molecular patterns associated with the parasites and derived from tissue damage occurs, leading to increased expression of haem oxygenase [6]. Platelets have a undefined role in the pathophisioloy of malaria [28, 29]. But in recent years, studies showed that inhibition of platelets activation correlates with decreased pulmonary vascular permeability due malaria infection [30, 31]. Also, it has already been observed that the increase in HO-1 expression correlates with decreased platelet activation and adhesiveness [32, 33]. Therefore, the hypothesis of this work that the modulation of haem oxygenase activity alters thrombocytopaenia and lung injury during infection with *Plasmodium berghei* strain NK65.

## Methods

### Mouse models of malaria

Wild Type C57BL/6 weighing 20–25 g was obtained from the Oswaldo Cruz Foundation breeding unit and used throughout the study. All animals used in this work were male and six to eigth weeks old. The animals were kept at constant temperature (25 °C) with free access to food and water in a room with a 12-h light/dark cycle. *Plasmodium berghei* strain NK65 was kindly provided by Dr. Juliana Tavares (UFMG). Mice were infected intraperitoneally (i.p.) by injection of 200 µL of 1× phosphate buffered saline (PBS) with 10^4^ parasitized red blood cells (pRBC [34]) or, as control group, 10^4^ red blood cell (RBC). Parasitaemia was measured from the count of parasitized and non-parasitized red blood cells in a total of 100 cells. This count was repeated in five independent fields and the average of the percentage of parasitized red blood cells performed. All analyses were performed at day 7–10 post-infection. Between 90 and 100% of infected mice developed lung injury.

### Ethics statement

The Animal Welfare Committee of the Oswaldo Cruz Institute approved the experiments in these studies under license number P-0528-08 and L025/15. The procedures described in this study were in accordance with the local guidelines and guidelines published in the National Institutes of Health Guide for the Care and Use of Laboratory Animals. The study is reported in accordance with the ARRIVE guidelines for reporting experiments involving animals.

### Treatment

For each experiment, a new solution of Cobalto Protoporphyrin IX/CoPPIX (PORPHIRIN-Logan, Utah) at a concentration of 5 mg/kg was prepared. The CoPPIX is used as an inducer of the haem oxigenase-1 enzyme [23]. The drugs were diluted in 0.9% sodium chloride/sterile saline (HEMAFARMA) solution containing 0.5% DMSO (Sigma). The solutions were sonicated (2510-BRANSON) for 20 min, and 200 μl was administered intraperitoneally for 8 days, starting at the day after infection. Saline solution with 0.5% DMSO (sigma) was administered (i.p.) as a group control. Infected and non-infected animals were evaluated for parasitaemia and survival until 15 days after the infection.

### Bronchoalveolar lavage fluid (BALF)

The animals were submitted to euthanasia by inhalation of isoflurane and bronchoalveolar lavage (BAL) samples were collected. After euthanasia, the trachea and section of adjacent muscles were exposed. Thereafter, a small hole was opened in the prominent region of the tracheal cartilage for insertion of a cannula attached to a 21G (BD) needle. Bronchoalveolar lavage fluid (BALF) was obtained by injecting 1 mL of ice-cold PBS, followed by aspiration of the contents, and this procedure was repeated 3 times. At the end of the process, an approximate volume of 0.9 mL of BALF per mouse was recovered. BALF was used for total and differential leukocyte count and for protein measurement. Total leukocytes (diluted in Turk’s 2% acetic acid fluid) were counted using Neubauer chamber haemocytometer. Differential counts were performed in cytospins (Cytospin3, Shandon, CA, USA) stained by the May-Grünwald-Giemsa method. The BALF was spun at 350 g at room temperature for 5 min, and the supernatant was removed and stored at − 80 °C for further analyses. BALF total protein concentration was measured using a BCA protein assay kit (Thermo Scientific, Waltham, MA, USA).

### Cytokine determinations

Perfused lungs from infected and uninfected mice were excised and homogenized in 750 mL of a protease inhibitor cocktail (Complete, mini EDTA-free Roche Applied Science, Mannheim, Germany) for 30 s, using a Ultra-Turrax Disperser T-10 basic (IKA-Guangzhou, China). Homogenates were stored at − 20 °C, for analysis of cytokines using a commercial ELISA kit according to the manufacturer’s instructions (R&D Systems Duo set kits, Minneapolis, USA).

### Lung permeability

The lung permeability was determined by intravenous injection of Evans blue dye (Sigma-Aldrich Brasil LTDA, São Paulo, Brazil) 2% (w/v) solution in phosphate-buffered saline (PBS). One hour later the animals were sacrificed with terminal anesthesia by isoflurane and the vasculature was intracardiacaly perfused with 20 mL phosphate-buffered saline (PBS) using a peristaltic pump system. The lungs were collected and placed in 2 mL of formamide (VETEC Química Fina LTDA, Duque de Caxias, RJ, Brazil) at 37 °C, overnight to extract Evans blue dye from the tissue. Absorbance was measured at λ = 620 nm (Molecular Devices Spectra Max 190, Sunnyvalle, CA, USA). Evans blue dye concentration was calculated from a standard curve and was expressed as mg of Evans blue dye per lung tissue.

### Lung histology

Perfused lungs from infected and uninfected mice were inflated by injecting 1.0 mL of 10% buffered formalin through the same catheter used to perform BALF. Lungs were removed fixed in formalin and embedded in paraffin. Lungs sections of 5-µm thickness were stained with haematoxylin–eosin. Analysis of tissue sections was performed in an Olympus BX41 microscope (Melville, NY, USA) at a magnification of 200×. The number of mononuclear cells in lung tissue was determined by the pointcounting technique across 20 random, non-coincidents microscopic fields in an Olympus BX41 microscope at a magnification of 1000×.

### Platelet count

A small amount of peripheral blood (2 µL) was obtained from the tail vein. The blood was immediately transferred to a solution containing 47 μL of buffered HT solution (10 mM Hepes, 137 mM NaCl, 2.8 mM KCl, 1 mM MgCl 2, 12 mM NaHCO 3, 0.4 mM Na 2 HPO 4, bovine serum albumin (Citric acid 8 g/L, sodium citrate 22.4 g/L, glucose 2 g/L, sodium citrate; pH 5.1) and 0.5 μl of APC fluorophore anti-CD41a antibody (clone eBioMWReg30 eBioscience), followed by incubation for 30 min. After incubation, 250 μl of the lysis solution (FACS lysing-BD) was added, and incubated for another 10 min at room temperature, and then stored 4 °C. To perform platelet counts 40 µL of beads (SPHEROTM AccuCount Particles-Spherotech, Inc.) with known size and 40 µL of blood were used. The beads are larger than the platelets and do not present fluorescence, thus allowing the identification and quantification of the platelets. The acquisition was performed using the flow cytometer (FacsCalibur, BD), and analysed using the Cell Quest (BD) software.

### Measurement of platelet-leukocyte aggregate formation

One hundred μL of peripheral blood was obtained by cardiac puncture using a 21G needle containing 20 μL of an anticoagulant solution composed of PSG buffer (5 mM PIPES, 145 mM NaCl, glucose 5.5 mM (Hepamaxs-Blau) and 0.5 mM prostaglandin E1 (PGE1, Cayman Chemical). Platelets were labelled with 0.5 µL of APC fluorophore labelled anti-CD41a (clone: eBioMWReg30 eBioscience); monocytes with 0.8 µL of labelled anti-CD14 antibody (clone: rmC5-3 BD Bioscience) with PE fluorophore, red cells with 0.8 µL of labelled anti-TER119 antibody (clone:TER-119 Biolegend) with PE fluorophore, and neutrophils with anti- Ly6G (clone: 1A8 BD Bioscience) labelled with PE fluorophore and 0.6 µL labelled anti-CD11b (clone: M1/70 eBioscience) with PE-Cy7 fluorophore. The following control isotypes were used in this work: 0.5 µL of mouse IgG1 anti-mouse (clone: eBRG1 eBioscience) APC, 0.8 µL of IgG1 mouse anti-mouse PE (clone: RTK2758 BD Pharmingen), and 0.6 µL of IgG2b mouse anti-mouse (clone: eB149/10HS eBioscience) PE-Cy ™ 7. The samples were incubated for 30 min, protected from light. After incubation, 250 μl of the lysing solution (FACS lysing-BD) was added, incubated for a further 10 min at room temperature, and storage in the refrigerator until read on the flow cytometer (FacsCalibur, BD). The analyses were performed using the Cell Quest (BD) program.

### Western blotting

Macerated lungs (40 µg of total proteins) were diluted in Laemmli buffer. The proteins were then denatured by heating at 100° C for 10 min and then applied to the gel. Laemmli 4× buffer consisted of 1 M Trizma Base (Sigma) solution pH 6.8, 8% SDS, 40% glycerol (Invitrogen), 20% β-mercaptoethanol (Sigma), bromophenol blue (Sigma) and water Milli-Q. The samples were electrophoresed (12% acrylamide gel) with the application of 125 V for one and a half hours and then transferred to nitrocellulose membrane (GE Healthcare). The transfer was performed at 100 V for 50 min in a wet system (BioRad). After transfer, the membrane was incubated for 1 h with 5% milk blocking solution in TBS/T (addition of 0.05% Tween 20, Sigma). Subsequently, the membrane was washed with TBS/T and incubated overnight with rabbit monoclonal antibody against mouse anti-VCAM-1 (abcam), at 1:1000 dilution in 5% skim milk in TBS/T. On the second day, the membrane was washed three times for 5 min with TBS/T and incubated for one and a half hours with anti-peroxidase-conjugated rabbit anti-IgG antibody (Vector) diluted 10,000-fold. The membrane was then washed three times for 5 min with TBS without Tween^®^ and immersed in a solution composed of equal parts of SuperSignal West Pico Stable Peroxide Solution (Pierce) and SuperSignal West Peak Luminol/Enhancer Solution (Pierce) for 20 min. After this time, excess solution was removed and the membrane was enclosed in a cassette containing a photosensitive film (Kodak) for development.

The loading control was done by the detection of β-actin expression. The membranes were washed three times for 5 min with TBS/T, then incubated for 15 min in the stripping solution (Unisciense), then washed three more times for 5 min with TBS/T. The membranes were then blocked for 30 min and β-actin antibody primary rabbit anti-mouse (Sigma) was added for 30 min at room temperature on the shaker (Labline). After this period three washes were again performed for 5 min with TBS/T, followed by incubation for 30 min with goat anti-rabbit IgG coupled to fluorophore (IRDye 800-RD) diluted 20,000-fold and the development in the Odssey Clx unit (Uniscience).

### Statistics

Statistical analysis was carried out using the GraphPad Prism software (San Diego, CA, USA). P values were calculated using an unpaired Ordinary one-way ANOVA test when results from more than two groups were analysed among each other. P values were generated using an unpaired T-test when result from only two groups were analysed. In addition, Shapiro–wilk normality test was performed. Results are expressed as mean ± SEM (median (IQR)). The level of significance was set at P​​ ≤ 0.05.

## Results

### *Plasmodium berghei* infection induces platelet activation and thrombocytopaenia

**Fig. 1:**
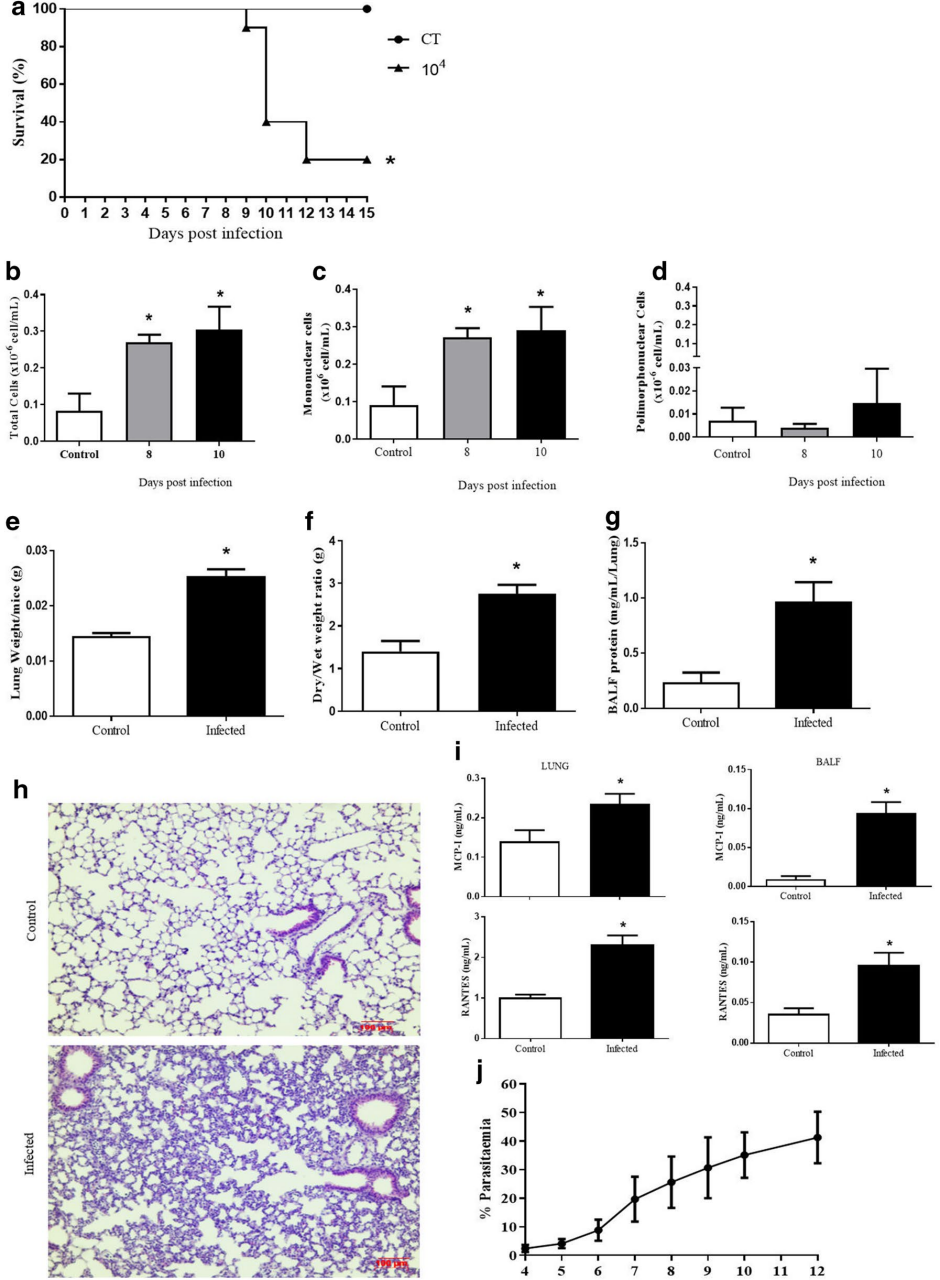
*Plasmodium berghei* strain NK65 infection induces pulmonary injury. C57B1/6 animals were inoculated i.p. with 10^4^ non-parasitized red blood cells (NP-RBC) or with 10^4^ parasitized red blood cells (PRBC). **a** Survival curve during 15 days. In a different set of experiments the animals were euthanized after 8 days. BALF was collected and the total and differential number of cells counted. The total cell count was performed on the Turk solution, and differential counts were performed in cytospin smears stained by Panótico. **b** Number of total cells Number of mononuclear cells. **c** Number of polymorphonuclear cells. **d** For evaluation of lung oedema and histological studies animals were euthanized 9 days after infection. **e** Total lung weight. Ratio between dry/wet weight. **f** Protein concentration in the BALF. **g** Histological analysis. **h** The sections were stained with haematoxylin and eosin. The patterns shown in the photos are representative of three experiments, each containing five animals. Magnification of ×10. The bar on the lower right corner of the photomicrographs indicates 100 µm. BALF and lung tissues were also processed for ELISA. **i** CCL-2/MCP-1, CCL-5/RANTES, both evaluated in lung tissue (left side graphic) and in BALF (right side graphic). **j** Parasitaemia during 12 days. Graphics are representative of three experiments, each line being the average of ten animals per experimental group; *p ≤ 0.05 when compared to uninfected controls. P values were generated using an unpaired T-test

Van den Steen et al. [26] demonstrated the development of experimental malaria-associated lung injury, with a survival rate of around 10% after 12 days. Here, using the same model, it was observed that infected animals start dying on the ninth day of infection. The highest mortality was seen between 9 and 12 days after infection, with a survival rate at the end of the observation period being 20% (Fig. 1a). Besides, an intense accumulation of leukocytes in the BALF on days eight and ten after infection (Fig. 1b–d) was observed. Differential leukocytes count showed that the predominant cell leukocyte accumulated was mononuclear cells (Fig. 1c), while the number of polymorphonuclear cells did not change significantly when compared to uninfected animals (Fig. 1d). Here using this animal model, also observed a significant increase of oedema formation (Fig. 1e–g), an intense inflammatory infiltrate in the pulmonary parenchyma (Fig. 1h) and the release of important chemokines in the pulmonary lysate (Fig. 1i) after 9 days post-nfection. Figure 1j observed the animals’ parasitaemia curve over the days after infection. The analyses were carried out on the 9th day after the infection as it is the point at which the mortality of the animals begins.

**Fig. 2:**
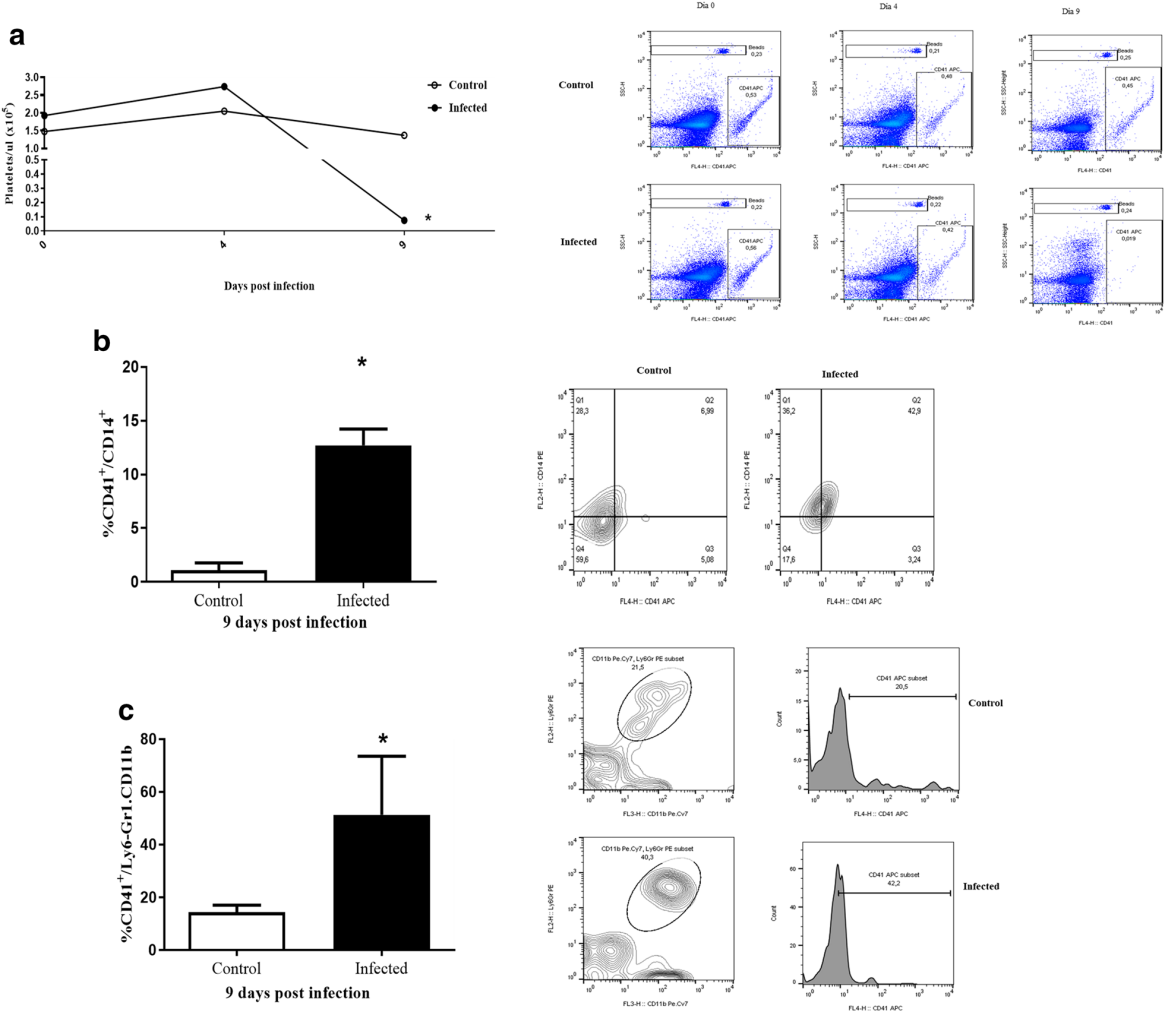
*Plasmodium berghei* strain NK65 infection induces platelet activation. **a** Number of circulating platelets was analysed on day one, four and nine after infection. Blood was drawn from the tail vein and labelled with CD41^+^ antibody for platelet identification. Specified amount of beads, of known size were added for quantification of platelets by cytometry. **b**, **c** were analysed 9 days after infection. **b** Analysis of platelet/monocytes heteroaggregates by labelling with CD41^+^ and CD14^+^, respectively. **c** Analysis of platelet/neutrophils heteroaggregates by labelling with CD41^+^ and Ly6-Gr1.CD14^+^, respectively. Each bar composed of five animals, representative of five experiments where *p ≤ 0.05 when compared to uninfected control. P values were generated using an unpaired T-test

Thrombocytopaenia is a frequent haematological manifestation in malaria, caused by both for *P. vivax* and *P. falciparum* [35]. To evaluate the occurence of thrombocytopaenia in during experimental malaria infection, peripheral blood was collected from infected and control animals for haematological analysis using flow cytometry analysis. Figure 2a shows that control animals exhibited a relatively equal number of platelets over the observation period. However, the platelet counts of infected animals significantly decreased on the ninth day of infection. This result indicates that this animal model reproduces characteristics of the human disease at least with regard to the occurrence of thrombocytopaenia.

One potential explanation for thrombocytopaenia in infected animals is formation of cellular aggregates. Flow cytometry was used to evaluate formation of platelets-monocytes (Fig. 2b) and platelets-neutrophils (Fig. 2c) aggregates on the ninth day of infection. Results here presented have been demonstrated that *P. berghei* infection causes activation of platelets and subsequent formation heteroaggregates of platelets and leukocytes, contributing to the underestimation of the number of platelets in the peripheral count.

### HO-1 activity protects animals from mortality by *P. berghei* infection without affecting parasitaemia

**Fig. 3:**
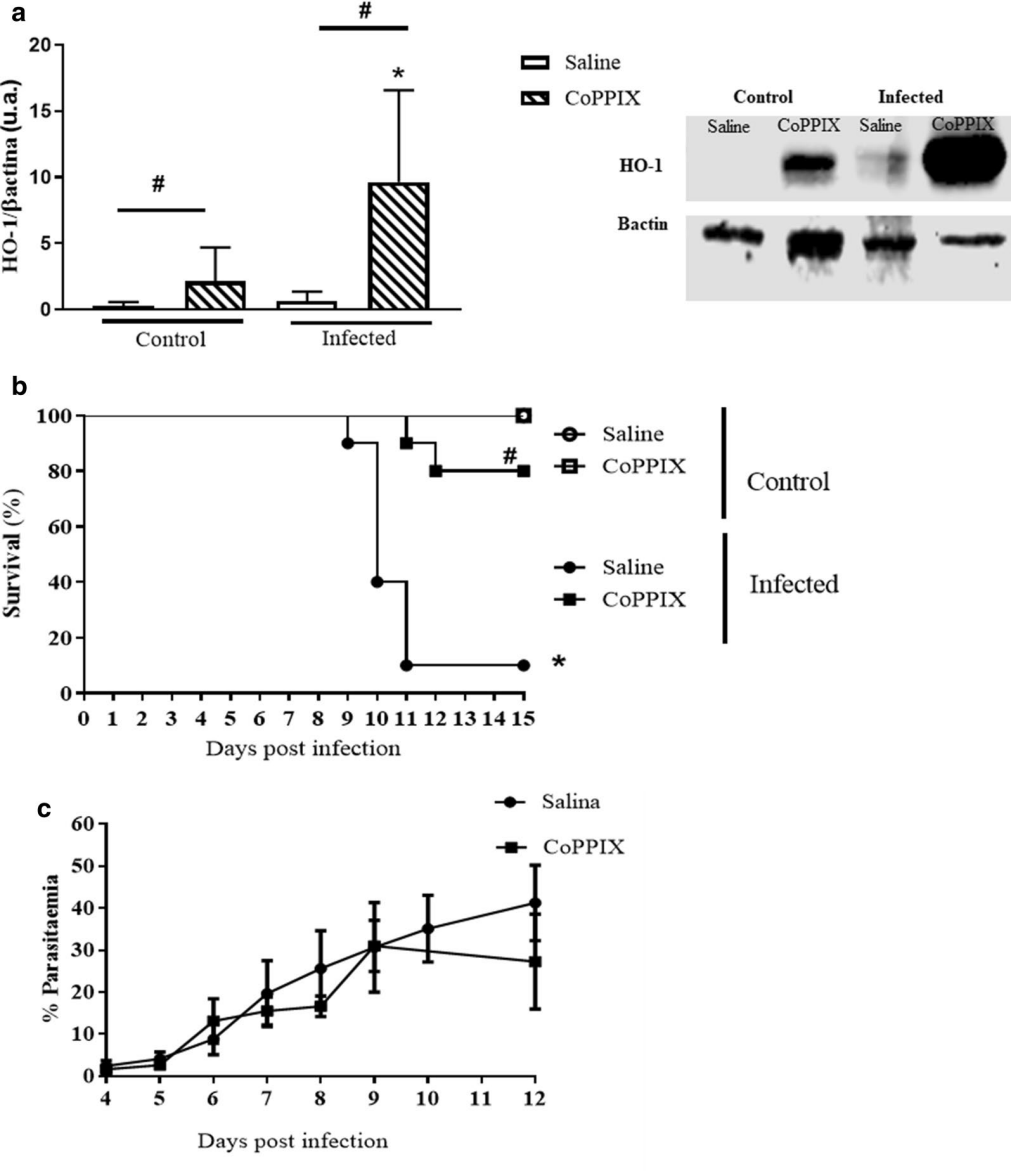
Effect of CoPPIX on the development of malarial infection. Animals received daily i.p. injection of saline with 0.5% DMSO (Saline) or 5 mg/kg of CoPPIX (CoPPIX). Blood smears were used to analyse the parasitaemia. **a** HO-1 expression increased after CoPPIX treatment, after 9 days infection. **b** Survival curve during 15 days. **c** Parasitaemia during 12 days, representative of three experiments, where *p ≤ 0.05 when compared to infected animals in relation to uninfected controls, and (#) indicates p ≤ 0.05 infected animals treated with CoPPIX in relation to infected animals treated with saline. P values were calculated using an unpaired Ordinary one-way ANOVA test

Modulation of the haem oxygenase-1 (HO-1) enzyme in experimental cerebral malaria [23] and pulmonary injury [26] was shown to increase survival. To further examine HO-1 in pulmonary malaria, protoporphyrin cobalt (CoPPIX) as an inducer of HO-1 activity in vivo was used [23]. The treatment started 1 day after infection and was administered daily at 5 mg/kg CoPPIX for 8 consecutive days. Treatment with CoPPIX leads to an increase in the expression of the HO-1 enzyme in uninfected animals and this increase becomes more significant once infection occurs (Fig. 3a). Saline-treated animals exhibited extensive mortality started 9 days post-infection (Fig. 3a). However, only 12% of the infected animals treated with CoPPIX died between the eleventh and twelfth day of infection (Fig. 3b). Importantly, CoPPIX treatment did not alter parasitaemia in the infected group (Fig. 3c). These results indicate that treatment with CoPPIX has a protective effect in experimental malaria, increasing survival by a mechanism that is independent from the control of parasite replication and is probably related to modulation of the host response to infection.

### Increased HO-1 activity prevents alveolar-capillary barrier disruption in infected animals

**Fig. 4:**
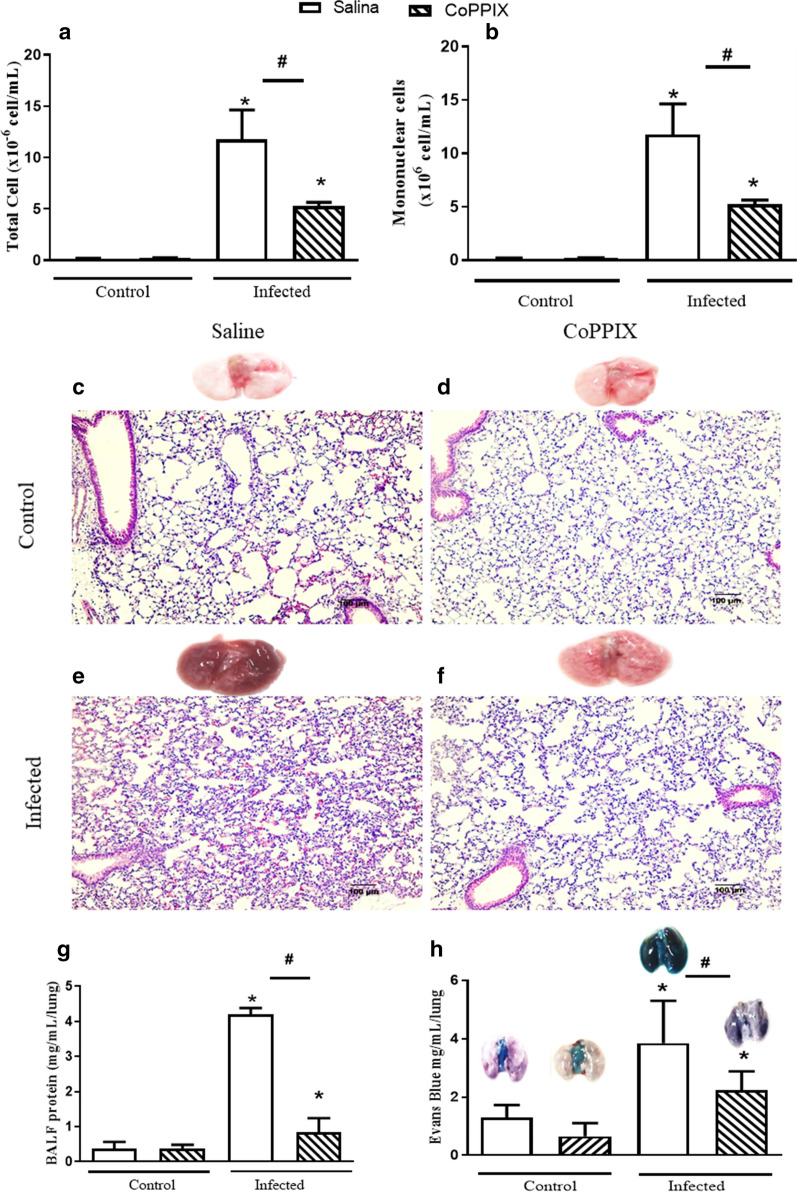
Treatment with CoPPIX decreases lung pathology. Animals received daily i.p. injection of saline with 0.5% DMSO (Saline), or 5 mg/kg CoPPIX (CoPPIX) and were euthanized 9 days after infection. **a** Total leukocyte counts. **b**, **c** represent differential leukocyte counts. Mononuclear cells (**b**) and polymorphonuclear cells (**c**). Each bar composed of five animals. **d**–**g**. Macroscopic aspect of lungs and photomicrographs from control and infected animals treated or not with CoPPIX. Photos are representative of five animals per experimental group, and three independent experiments. Magnification of ×10. The bar on the lower right corner of the photomicrographs indicates 100 µm. In H, protein content in the BALF. In I, Evans blue extravasation to the lung tissue (with macroscopic photos of the lungs representative of each group). Each bar averaging to 8–10 animals. The experiments were repeated 3 times with similar results. *p ≤ 0.05 when compared to uninfected controls, and (#) indicates p ≤ 0.05 when compared to animals treated with CoPPIX. P values were calculated using an unpaired Ordinary one-way ANOVA test

To investigate whether COPPIX treatment influences host response, cell migration into the lungs was evaluated. CoPPIX treatment was associated with a significant reduction in leukocyte numbers in the BALF as compared to infected animals treated with vehicle (Fig. 4a). The reduction in leukocyte numbers was mainly in the numbers of mononuclear cells as demonstrated by differential cell counting (Fig. 4b). Similarly, histologic analysis revealed that treatment with CoPPIX decreased cell infiltration in the lung parenchyma (Fig. 4f) when compared to infected animals treated with saline (Fig. 4e). It was also observed that treatment with CoPPIX does not lead to any lung damage since there are no noticeable differences between the lungs of animals that are not infected and treated with saline (Fig. 4c) or CoPPIX (Fig. 4d). Changes in the lung parenchyma of CoPPIX treated animals were also obvious by macroscopic examination. Lungs from CoPPIX treated animals showed a less intense dark coloration indicating a milder inflammation (Fig. 4f).

Infected animals had a significant extravasation of protein into BAL fluid relative to their uninfected controls (Fig. 1g). However, infected animals treated with CoPPIX showed significantly less extravasation compared to infected groups treated with saline indicating that treatment with CoPPIX protects against alveolar-capillary barrier dysruption. The Evans blue method was used to confirm disrupted alveolar-capilary barriers and that treatment with CoPPIX prevented this event (Fig. 4f).

### Increased HO-1 activity decreases the expression of VCAM-1 but does not inhibit chemokine release

**Fig. 5:**
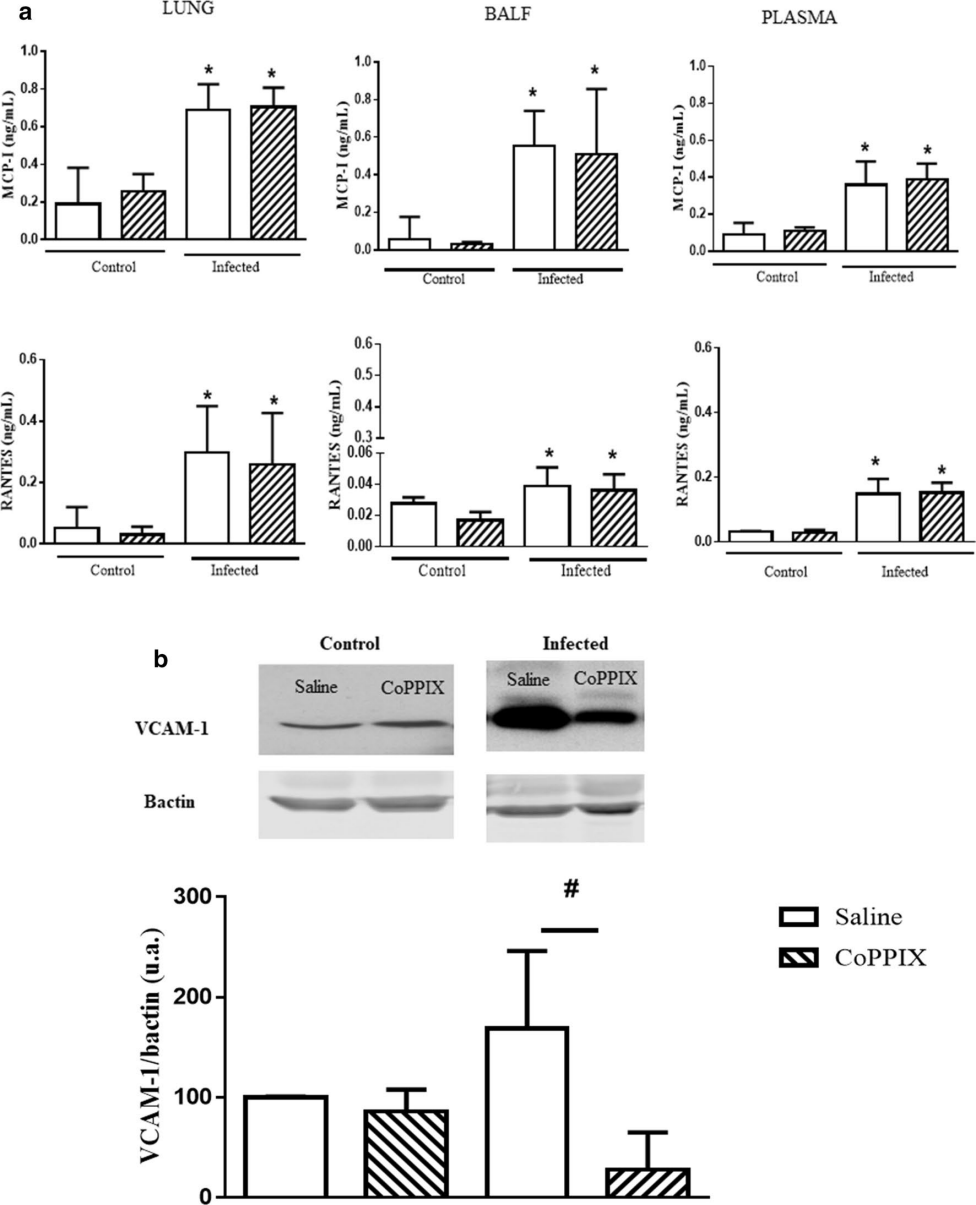
Effect of CoPPIX on chemokines release and expression of VCAM-1. Animals received daily i.p. injection of saline with 0.5% DMSO (Saline) or 5 mg/kg CoPPIX (CoPPIX) and were euthanized 9 days after infection. The lungs, BALF and peripheral blood were collected and processed for ELISA. A. CCL-2/MCP-1, and CCL-5/RANTES in lungs (left panels), BAL (middle panels) and plasma (right panels). Each is the mean from 5 animals. The experiment was repeated three times with similar results. *p ≤ 0.05 when compared to uninfected controls. **b** Lung samples from control and infected animals treated or not with CoPPIX were collected and used for analysis for VCAM-1 expression by western blot. β-bactin was used as normalizer (upper panel). Densitometry analysis is shown in the bottom panel. This is representative of five animals. (#) indicates p ≤ 0.05 when compared to CoPPIX treated animals. P values were calculated using an unpaired Ordinary one-way ANOVA test

To further investigate the modulatory effect of HO-1 in lung inflammatory reaction of infected animals the release of chemokines in the lung tissue, BALF and plasma 9 days after infection were measured. As expected, a significant increase in CCL-2/MCP-I (Fig. 5a top panel) and CCL-5/RANTES levels (Fig. 5a bottom panel) in all the analysed sites were observed. Surprisingly, treatment with CoPPIX did not interfere with the release of these chemokines in any of the compartments analysed.

Because endothelial activation influences cell migration and is also modulated by the activation of HO-1 [36], used lung lysates to evaluate the expression of VCAM-1 western blot. The infected animals treated with CoPPIX showed significantly less VCAM-1 expression than infected groups treated with vehicle (Fig. 5b). This indicated lesser activation of the endothelial cells, a potential explanation for lesser inflammation found in the treated animals.

### Increased HO-1 activity prevents platelets activation and thrombocytopaenia induced by *P. berghei*

**Fig. 6:**
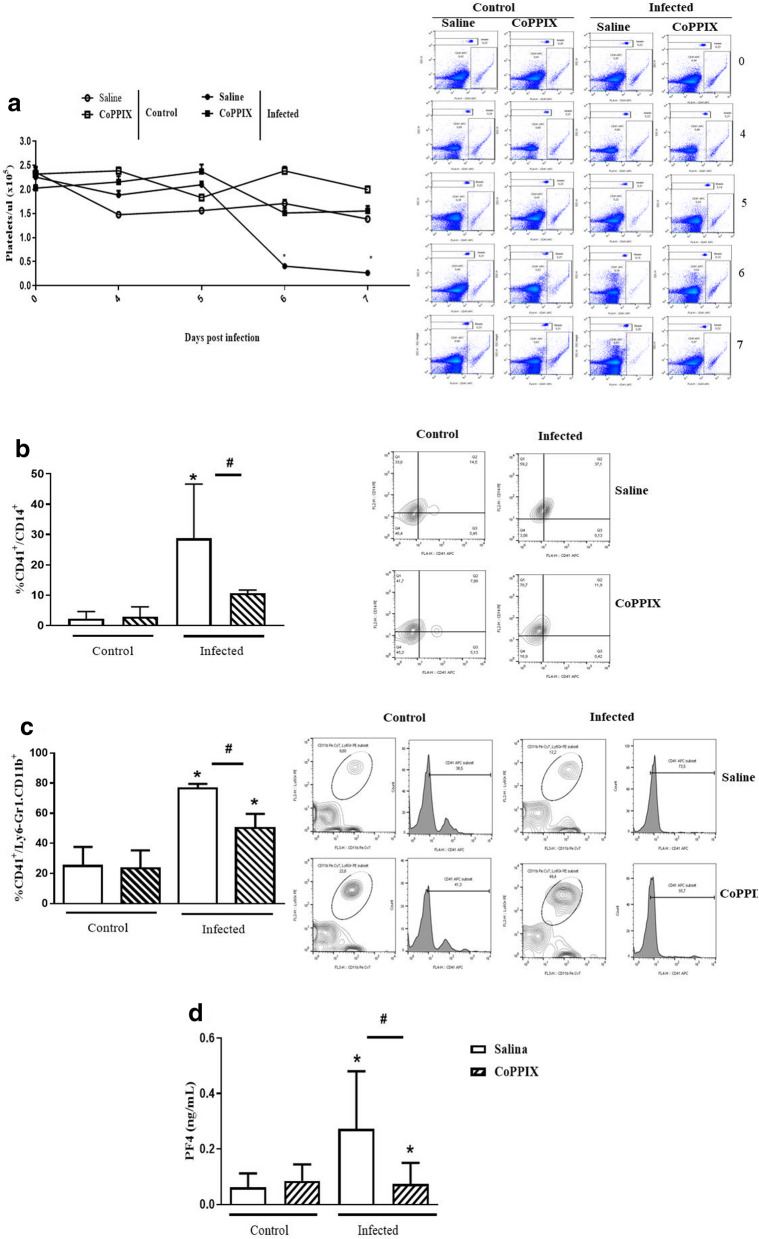
CoPPIX treatment decreases platelet activation in *P. berghei* NK65 infected animals. C57B1/6 animals were inoculated i.p. with 10^4^ non-parasitized red blood cells (NP-RBC) or with 10^4^ parasitized red blood cells (PRBC). Animals received daily i.p. injection of saline with 0.5% DMSO (Saline) or 5 mg/kg CoPPIX (CoPPIX). Blood was collected from the tail vein at various times throughout the experiment. A. Platelet counts over a 7-day period. At day 7, cardiac puncture was performed and the blood was processed for cytometry. B. Analysis of the formation of platelet/monocytes heteroaggregates, by labelling platelets with CD41a^+^ and monocytes with CD14. C. Analysis of the formation of platelet/neutrophils heteroaggregates by labelling platelets with CD41a^+^ and neutrophils with Ly6-Gr1.CD11b^+^. D. BALF was collected processed for PF4 detection by ELISA. Each bar is the mean from 5 animals. *p ≤ 0.05 when compared to uninfected controls, and (#) indicates p ≤ 0.05 when compared to CoPPIX treated animals. P values were calculated using an unpaired Ordinary one-way ANOVA test

Control animals maintained a relatively constant platelet count throughout the experiment (Fig. 6a). As described above infected animals showed a decrease in the number of platelets by day six post-infection (Fig. 6a). Conversely, infected animals treated with CoPPIX did not developed thrombocytopaenia at any time during the experiment (Fig. 6a).

Infected animals showed increased formation of platelet-monocyte (Fig. 6b) and platelet-neutrophil (Fig. 6c) aggregates, demonstrating platelet activation [13]. Platelet factor 4 (PF-4), a platelet-derived CXC chemokine, was also increased in these animals (Fig. 6d), further suggesting platelet activation. On the other hand, infected animals treated with CoPPIX did not show significant increases in platelet heteroaggregates or PF-4 levels (Fig. 6b–d), indicating that HO-1 influences platelet activation and formation of aggregates of platelets and leukocytes in experimental malaria.

## Discussion

Pulmonary injury characteristically occurs after initiation of anti-malarial treatment and is considered a complication linked to the exacerbated inflammatory response [3]. Malaria-induced acute lung injury presents with alveolar-capillary inflammation, altered lung ventilation/perfusion relationships and gas exchange, variable airway obstruction, and increased pulmonary phagocytic activity [4]. In human and experimental malaria, metabolic changes, alteration of cytokine expression and sequestration of cells in the microcirculation of the brain and other organs are commonly seen [2, 37, 38]. One of the most important steps in the infectious process is the immunoinflammatory response that involves, among other events, the migration of cells and accumulation into the tissue. In this context, platelets are cells that have received much attention as new inflammatory roles have recently been described [11, 39]. However, their role and activities in malaria have been controversial at best. Previous studies showed that platelet depletion or inhibition of aggregation in infection with *P. berghei* strain ANKA leads to a longer survival, suggesting that role platelet the formation of cerebral or lung injury due malaria [30, 40]. Also, in experimental cerebral malaria, a lower incidence of cerebral malaria was observed when P-selectin deficient mice were used [41]. Regarding lung injury triggered by infection with *P. berghei* strain NK65, it has also been noted that von Willebrand factor (VWF—a glycoprotein synthesized in endothelial cells and megakaryocytes, what is crucial in normal haemostasis and thromboinflammation), contributed to increased alveolar leakage and VWF deficiency was associated with elevated parasite load, anaemia, and shortened survival time [31]. Here a possible relationship between platelet activation, pulmonary injury and thrombocytopaenia due to infection by *P. berghei* NK65 and examined the influence of haem oxygenase in platelet activation and acute pulmonary injury during *P. berghei* infection was investigated.

Thrombocytopaenia is a common complication in both vivax and falciparum malaria, but its causes are still not fully elucidated [35, 42]. Thrombocytopaenia in malaria seems to be correlated with platelet activation [35, 43]. The thrombocytopaenia was detected on the sixth day post-infection and that it correlated with the formation of heteroaggregates of platelet and leukocytes. Platelet adhesion to the endothelium or thrombopoiesis as contributors to thrombocytopaenia could not be excluded. Platelet responses are also altered in other infections syndromes. For example, platelet activation and aggregation are correlated with increased severity of human and experimental sepsis [44, 45], clinical and experimental studies shown that infection by Streptococcus induces platelet activation and increased adhesiveness that correlates to a worsening of the inflammatory picture [46].

Formation of heteroagregates of platelets and leukocytes, mainly monocytes, is a relevant pathophysiologic event in diseases such as diabetes [47], dengue [48], HIV infection [49], cardiovascular diseases [50] and in cancer [51]. Activation of platelets leads to increased adhesion to and activation of leukocytes, triggering the production and/or release of cytokines and chemokines. In fact, heteroaggregates of platelets and neutrophils were reported as deleterious in several models of pulmonary inflammation. In acid-induced lung injury heteroaggregates of platelets and neutrophils are frequently formed, and blockade of P-selectin leads to an improvement in the inflammatory response [14]. Also, neutrophil-dependent sequestration of platelets in the lung was identified in the transfusion-related lung injury (TRALI) model [52]. Worth mentioning the formation of platelet-induced neutrophil extracellular traps (NET) in the lung of animals with TRALI has also been described [53]. Data here presented suggest that similar events of platelet-mediated leukocyte activation may be present in *P. berghei* infection, contributing to a poor prognosis.

Malaria infection is accompanied by an intense release of haem after rupture of parasitized red blood cells. The presence of free haem during pathologic conditions triggers the induction of the enzyme haem oxygenase-1 (HO-1), which catalyzes haem releasing carbon monoxide and biliverdin, both potent antioxidant agents that may aid in the control of tissue damage. It was observed that treatment with CoPPIX did not interfere with the development of infection, however, CoPPIX treatment led to a significant increase in survival. Induction of HO-1 activity, in most cases by CoPPIX treatment, is associated to a protective effect in different pathologic conditions such as cardiac transplant rejection [54], and abortion after infection by *Brucella abortus* [55].

In experimental cerebral malaria induced by infection with *P. berghei* strain ANKA statin treatment has been shown to increase expression of the HO-1 and survival rate [56]. Similarly, CoPPIX treatment also increased survival in *P. berghei* ANKA infection [23], while haem, an important inducer of HO-1, decreased mortality in a model of pulmonary malaria induced by *P. berghei* NK65 in DBA/2 animals [26]. Once no alterations were observed in the development of parasitaemia after CoPPIX treatment it can be possible that the protective effect of CoPPIX is a consequence of the control of the inflammatory response. In fact, CoPPIX treatment decreased oedema and accumulation of cells in the BAL and the lung parenchyma. Similar findings were reported in an influenza A virus model of infection where administration of haem (as HO-1 inducer) decreased lung oedema and cell accumulation into the lung parenchyma [57].

Although CoPPIX inhibited oedema and cell accumulation in experimental model here developed it did not interfere with the release of CCL-2/MCP-1 and CCL-5/RANTES. In contrast, the role of HO-1 in the malaria-associated lung injury model was evaluated in *P. berghei* ANKA-infected DBA/2 mice. Decreased accumulation of cytokines IFN-γ, IL-10 and CCL-2/MCP-1 in the lung and serum were found [26]. These results are in contrast with ours, but it is important to note that the authors used a different strain of *Plasmodium* and a different mouse background. Furthermore, they looked at cytokine expression by cytometry while detected the released protein by ELISA. However additional work analyzing a wider range of cytokines and examining both RNA and protein expression is necessary to gain a more definitive picture about cytokine modulation by HO-1 in malaria ARDS.

Despite the lack of effect of CoPPIX on cytokine release, activation of endothelial was significantly affected as indicated by the decrease in the expression of VCAM-1 in CoPPIX-treated animals. This corroborates with the inhibition of oedema and cell accumulation, events associated with endothelial cell activation, and suggests that increased expression of HO-1 has cytoprotective and anti-inflammatory effects reducing activation of endothelial cells and consequently the expression of adhesion molecules, cell migration and oedema. This effect of HO-1 is not restricted to malaria and positive regulation of HO-1 decreases the expression of adhesion molecules, such as P-selectin and E-selectin, and leukocyte adhesion in a model of endotoxic shock [58].

Interestingly, a case study of a HMOX1-deficient patient demonstrated that the absence of the enzyme triggered endothelial damage, high plasma levels of von Willebrand factor, increased ICAM-1 expression, coagulation and fibrinolysis defects, generalized inflammation and premature atherosclerosis. The patient died at 6 years old suffering from growth retardation, anaemia, leukocytosis, thrombocytosis, coagulation abnormality, elevated serum levels of haptoglobin, ferritin and haem, a low serum bilirubin concentration and hyperlipidaemia. Autopsy revealed the presence of amyloid deposits, foamy macrophages, fatty streaks and fibrous plaques [59]. This case of HO-1 deficiency is an example that confirms the importance of HO expression in homeostasis and its involvement with activation of endothelial cells and the expression of adhesion molecules.

When analysing the role of HO-1 in thrombocytopaenia, it was observed that infected animals treated with CoPPIX did not show a decreased in peripheral platelet counts. In addition, platelet activation was inhibited as demonstrated by a significant decrease in formation of platelet/leukocytes heteroaggregates. It is documented in the literature that the HO-1 enzyme plays an important role in inhibiting platelet activation and aggregation under stress conditions [33], in cardiovascular diseases [32] and in hepatic injury caused by ischemia and reperfusion events [60]. In the present work, reduced platelet activation was confirmed by quantifying the release of an important platelet-derived immunoregulatory mediator, PF4. Systemic treatment with COPPIX dramatically decreased the release of this chemokine into the BAL fluid, consistent with reduced platelet activation and inflammation. These data add to previous studies that inflammation ans accumulation leukocytes in experimental influenza infection and in other ALI models [61, 62].These studies indicate that the activation of the HO-1 enzyme leads to decreased platelet activation and adhesiveness that could influence the peripheral platelet count.

Taking together, data here presented suggest that that HO-1 modulation may have a beneficial role in the treatment of lung injury triggered by malaria. Nevertheless in a very elegant literature review about the role of haem oxygenase-1 in experimental and human malaria, the authors made clear of the dual role of this enzyme, once in the asymptomatic phase (hepatic stage of the disease), HO-1 expression reduces inflammation and consequently provides a favorable environment for the development and multiplication of parasites. But in posterior stages of infection (blood stage), characterized by the accumulation of free haem and haemozoin as a result of parasitized erythrocyte burst and inflammatory response, HO-1 increase would be more beneficial as it would prevent the toxic effects of haem and reduce an exacerbated inflammation [63]. Since the liver stage can coexist with the blood stage in endemic areas where the rate of infection is high and an individual can be infected several times in a short period of time, understanding the HO-1 mechanisms at work during the different stages of infection by *Plasmodium* sp becomes of fundamental importance.

## Conclusion

The study demonstrates that haem oxygenase-1 is an important molecular effector that influences thrombocytopaenia and lung injury associated with malaria. CoPPIX treatment was able to decrease alveolar-capillary membrane “leak”, alveolar monocyte and macrophage accumulation, and lung oedema, indicating that HO-1 is a key determinant of critical pathophysiologic events in this model of malaria associated lung injury. Thrombocytopaenia is another important aspect of the pathophysiology of malaria. Few studies have been conducted to understand what leads to this phenomenon. In conclusion, during experimental malaria infection, there was a dramatic platelet activation demonstrated by the formation of heteroaggregates of platelets and leukocytes and by the release of PF-4, and that treatment with CoPPIX decreases this activation. These data suggest that a better understanding of the role of haem oxygenase in the various infectious stages of malaria, may be an important tool in the treatment of lung injury and thrombocytopaenia associated with malaria.

## Data Availability

The data supporting the conclusions of this article are provided within the article and its additional files. The original datasets analysed in this current study are available from the corresponding author upon request.

## Abbreviations

MA-ARDS: Malaria-associated acute respiratory distress syndrome
ARDS: Acute respiratory distress syndrome
RBC: Red blood cell
pRBC: Parasitized red blood cell
BALF: Bronchoalveolar lavage fluid
PF4: Platelet-derived chemokine (C-X-C motif) ligand 4(CXCL4)
